# Lessons Learned from Protective Immune Responses to Optimize Vaccines against Cryptosporidiosis

**DOI:** 10.3390/pathogens7010002

**Published:** 2017-12-24

**Authors:** Maxime W. Lemieux, Karine Sonzogni-Desautels, Momar Ndao

**Affiliations:** 1 National Reference Centre for Parasitology, Research Institute of the McGill University Health Centre, McGill University, Montreal, QC H4A 3J1, Canada; maxime.lemieux@mail.mcgill.ca (M.W.L.); karine.sonzogni-desautels@mail.mcgill.ca (K.S.-D.); 2 Department of Medicine, Division of Experimental Medicine, Faculty of Medicine, McGill University, Montreal, QC H4A 3J1, Canada; 3 Faculty of Agricultural and Environmental Sciences, Institute of Parasitology, McGill University, Ste-Anne-de-Bellevue, QC H9X 3V9, Canada; 4 Department of Medicine, Division of Infectious Diseases, Faculty of Medicine, McGill University, Montreal, QC H4A 3J1, Canada

**Keywords:** cryptosporidiosis, *Cryptosporidium*, immune response, infection, vaccine, innate immunity, mucosal immunity, adaptive immunity, T_H_1 immune response, T_H_2 immune response

## Abstract

In developing countries, cryptosporidiosis causes moderate-to-severe diarrhea and kills thousands of infants and toddlers annually. Drinking and recreational water contaminated with *Cryptosporidium* spp. oocysts has led to waterborne outbreaks in developed countries. A competent immune system is necessary to clear this parasitic infection. A better understanding of the immune responses required to prevent or limit infection by this protozoan parasite is the cornerstone of development of an effective vaccine. In this light, lessons learned from previously developed vaccines against *Cryptosporidium* spp. are at the foundation for development of better next-generation vaccines. In this review, we summarize the immune responses elicited by naturally and experimentally-induced *Cryptosporidium* spp. infection and by several experimental vaccines in various animal models. Our aim is to increase awareness about the immune responses that underlie protection against cryptosporidiosis and to encourage promotion of these immune responses as a key strategy for vaccine development. Innate and mucosal immunity will be addressed as well as adaptive immunity, with an emphasis on the balance between T_H_1/T_H_2 immune responses. Development of more effective vaccines against cryptosporidiosis is needed to prevent *Cryptosporidium* spp.-related deaths in infants and toddlers in developing countries.

## 1. Introduction

Among the causes of mortality worldwide, diarrheal-associated deaths are in the top 10 causes of mortality in humans and the fourth leading cause in children under 5 years of age (around 499,000 deaths every year) [1]. Human cryptosporidiosis caused by *Cryptosporidium hominis* and *C. parvum* is the second most common cause (following only rotavirus) of moderate-to-severe diarrhea in 0–11 month-old infants and the third most common in 12–23 month-old toddlers in sub-Saharan Africa and south Asia [2]. For example, in rural Bangladesh, 77% of children less than 2 years old were infected with *Cryptosporidium* spp. [3]. This infection was associated with failure to thrive and impaired cognitive functions in young children in developing countries [3,4,5]. More worryingly, around 202,000 deaths are attributable to cryptosporidiosis among children younger than 24 months old in sub-Saharan Africa, India, Pakistan, Bangladesh, Nepal and Afghanistan [6]. Among these deaths, around 59,000 are in excess in comparison if these children were *Cryptosporidium* spp.-negative [6]. *C. hominis* was isolated in 77.8% of cryptosporidiosis cases in children in sub-Saharan Africa and south Asia, with *C. parvum* present in 9.9% [6]. *C. parvum*-positive cases can arise from human-to-human transmission [6], but *C. parvum* is a zoonotic protozoan parasite and can also be transmitted from animal hosts to humans [7]. For this reason, veterinary students can sometimes get infected through contact with *C. parvum*-infected calves [8,9,10]. Also, *C. parvum* oocysts obtained from livestock can contaminate water [11], and *Cryptosporidium* spp. constitute a significant public health concern in developed and developing countries due to its ubiquitous nature [12]. *C. hominis* or *C. parvum* contamination of water can lead to foodborne outbreaks [7] following consumption of fruits and vegetables irrigated or washed with *Cryptosporidium* spp.-contaminated water [13]. Many waterborne outbreaks have been reported in developed countries following contamination with *C. hominis* or *C. parvum* oocysts of drinking (untreated surface water, water-treatment limitations, water-testing limitations, etc.) or recreational water (swimming pools, etc.) [14]. One of the most important waterborne outbreaks happened in Milwaukee, USA in 1993, in which 403,000 people showed symptoms of watery diarrhea following *Cryptosporidium* spp. infection [14,15] and many immunocompromised people died of the infection [16]. While the source of the infection is still debated [17], filtration system of Milwaukee’s water treatment plants was deficient and did not remove all oocysts from the treated water [15]. This outbreak cost USD 64.6 million in lost productivity and USD 31.7 million in medical costs [18]. Immunocompetent individuals usually recover spontaneously from a transient gastroenteritis characterized by watery diarrhea and abdominal cramps [7,15,19]. However, immunocompromised patients, such as HIV/AIDS patients, people under immunosuppressive treatments (cancer patients undergoing chemotherapy or patients with solid-organ transplants), patients with inheritable immunodeficiency syndromes (children with Severe Combined Immunodeficiency Syndrome (SCID)) and infants can develop chronic, severe and even life-threatening clinical signs [19]. AIDS patients were particularly at risk of lethal cryptosporidiosis before the availability of effective anti-retroviral therapies [20,21]. Recent advances in anti-retroviral therapy (ART) have markedly reduced the risk of cryptosporidiosis in HIV-infected individuals [22,23]. As a result, patients co-infected with HIV and *Cryptosporidium* spp. have much lower mortality rates compared to 15 years ago [24,25,26].

From a veterinary point of view, *Cryptosporidium* spp. infect a variety of mammals, including calves, dogs, cats, rabbits and birds [14,27]. Bovine cryptosporidiosis caused by *C. parvum* is a major problem in the dairy industry because infection is extremely prevalent [28,29,30] in newborn dairy calves and can cause life-threatening disease [31,32]. Because *C. parvum* is a zoonotic parasite and *Cryptosporidium* spp. complete the life cycle within a single individual [19], bovine cryptosporidiosis is also a public health concern. The risk is compounded by the fact that morbidity associated with infection in both humans and animals occurs at a very low parasite inoculum: 30 *C. parvum* oocysts are sufficient to cause a symptomatic human infection [33]. Despite extensive research on chemotherapy against *Cryptosporidium* spp., there is still a lack of curative treatments [34]. Moreover, no vaccine is available to prevent cryptosporidiosis in humans or animals, despite the urgent need [34]. The difficulty to develop efficient vaccines against *Cryptosporidium* spp. mostly comes from its unique life cycle which has already been reviewed [35]. Briefly, the oocysts excyst and sporozoites are released to infect intestinal epithelial cells (IECs) [36]. Sporozoites mature to become trophozoites and form type 1 meront (type 1 schizont) containing merozoite precursors [19]. Mature merozoites that become trophozoites complete the asexual cycle while those that become type 2 meronts continue to the sexual cycle of the parasite which will lead to production of infectious oocysts [19]. Because most stages of *Cryptosporidium* spp. life cycle occur inside IECs and are located intracellularly, but extracytoplasmically [19], surface proteins of sporozoites and merozoites are thought to be ideal candidate for vaccine development. However, the pathophysiology of cryptosporidiosis is not fully elucidated and developing an effective vaccine is a major challenge [37]. The aim of this review is to increase awareness of the immune responses that control *Cryptosporidium* spp. infection to be able to define appropriate targets in vaccine development.

## 2. Innate Immunity

**Figure 1:**
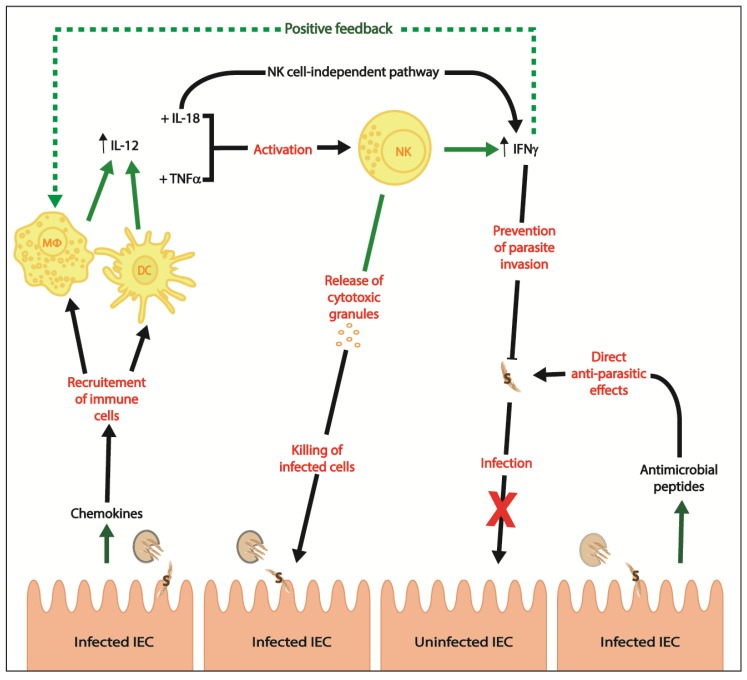
Innate immune responses during *Cryptosporidium* spp. infection. Green solid lines show release of molecules, green dotted line illustrates positive feedback, black solid lines present direction of effects and red wordings define effects. NK = NK cell, MΦ = macrophage, DC = dendritic cell, S = sporozoite and IEC = intestinal epithelial cell.

The critical role of the innate immune response to *C. parvum* infection has been covered elsewhere [38,39]. Briefly, innate immunity is required for controlling the intensity of *Cryptosporidium* spp. infection [36]. After ingestion of *Cryptosporidium* spp. oocysts, sporozoites are released in the intestinal lumen and migrate to IECs; while IECs are the first physical barrier against infection, they also are the main target for *Cryptosporidium* spp. sporozoites [19,36]. Chemokines are first released by *C. parvum*-infected IECs to promote chemotaxis at the site of infection; chemokines induce migration of dendritic cells in the ileum and the draining lymph nodes [40] (Figure 1). Inflammatory monocytes will also migrate to the subepithelial space in response to *C. parvum* infection and secrete TNFα and IL-1β [41]. These cytokines will increase permeability, therefore weakening the integrity of the intestinal epithelial barrier [41]. Also, nitric oxide NO is important in *C. parvum* infection clearance and reduces oocyst shedding in chronically infected nude mice [42]. NO is produced independently of IFNγ in *Cryptosporidium* spp. infections [43]. Inhibition of inducible nitric oxide synthase (iNOS) led to increased parasitism and oocyst shedding in *C. parvum*-infected piglets [44]. During *C. parvum* infection, the protective effect of iNOS depends on arginine availability in mice [42]. In fact, Leitch and He showed that supplementation with L-arginine decreases oocyst shedding in athymic nude mice [42]. IFNγ mediated production of chemokines by IECs recruits dendritic cells which clear *C. parvum* infection [39]. IECs also release antimicrobial peptides to destroy free parasites or can enter apoptosis if *C. parvum* infection already occurred [36] (Figure 1). Infection of IECs by *Cryptosporidium* spp. activates the MyD88 and NF-kB signalling cascade through Toll-like receptors (TLRs); in particular, TLR2 and TLR4 induce the production of human β-defensin 2 to help clear parasites [45,46].

Mucosal immunity is important for clearance of *Cryptosporidium* spp., as reviewed elsewhere [35]. The activation of antigen-presenting cells such as macrophages and dendritic cells is also important in *Cryptosporidium* spp. infections [47,48,49,50,51]. Dendritic cells can clear *Cryptosporidium* spp. from the site of infection via activation of adaptive immune responses [51,52]. But, dendritic cells, neutrophils and IFNγ are also important in *Cryptosporidium* spp. infection because they play a crucial role in pathogen recognition and clearance of the parasite through direct contact [50,51,53,54] (Figure 1). The crucial role of mucosal natural killer (NK) cells (non-T, non-B lymphocytes [55]) in *Cryptosporidium* spp. infection is an active field of research [56,57,58]. The important contributions of NK cells and IFNγ in innate immune responses against *C. parvum* infection have previously been reviewed [59]. Briefly, NK cells are an important source of IFNγ in cryptosporidiosis and they are key players in controlling the infection in mice [56,60]. In the acute phase of infection, *C. parvum* sporozoites induce production of IL-12 by macrophages and dendritic cells [61]. IL-12 acts synergistically with IL-18 and TNFα to activate NK cells [19,62,63] (Figure 1). Thus, the production of IFNγ by NK cells and macrophages in response to *Cryptosporidium* spp. is promoted by IL-12 and IL-18 [64,65]. Secreted IFNγ can inhibit *C. parvum* invasion and intracellular development by acting directly on enterocytes and preventing parasite invasion [50,54] (Figure 1). Moreover, NK cells can efficiently kill *Cryptosporidium* spp.-infected human IECs [58] by inducing programmed cell death via the action of released cytotoxic granules [62] (Figure 1).

Apart from its role in inducing IFNγ production by NK cells, IL-18 also has a NK cell-independent role (Figure 1). Rag2^−/−^gammac^−/−^ adult mice (deficient for NK, T and B cells) can clear *C. parvum* infection due to NK cell-independent IFNγ production [57]. In this case, IFNγ is probably produced by IL-18- and IL-12-activated macrophages [57]. However, NK, T and B cell-deficient Rag2^−/−^gammac^−/−^ adult or neonate mice have more severe *C. parvum* infections than T and B cell-deficient Rag2^−/−^ adult mice [56]. Consequently, even if both NK cell-dependent and -independent IFNγ have a protective role in innate immunity against *C. parvum* infection, presence of NK cells significantly helps to contain infection [56].

The susceptibility of interferon gamma receptor knock-out (IFNγR-KO) mice to *Cryptosporidium* spp. infection is an excellent example of the essential role of IFNγ for the control of cryptosporidiosis [66,67,68]. SCID-IFNγKO mice have heavier infections than SCID mice [69] and IL-12KO mice are highly susceptible to *Cryptosporidium* spp. infection [70,71]. In addition, treatment of newborn SCID mice with anti-IL-12 neutralizing antibodies exacerbates cryptosporidiosis [64].

## 3. Adaptive Immunity

### 3.1. Cell-Mediated Immune Responses

The innate response is important for initial control of *Cryptosporidium* spp. infection, but adaptive immune responses are required for resolution of this disease [19,36]. The gut-associated lymphoid tissue (GALT) of the intestine is the main line of defense against pathogenic and commensal organisms of the gastrointestinal tract [72]. The intestinal environment contains a very diverse pool of antigens from food and microorganisms [73]. The mucosal immune system is therefore an important barrier to protect against pathogenic organisms and to confer tolerance against food antigens and the gut microbiota [74]. The gut immune responses therefore encompass high numbers of pro-inflammatory cells to prevent infection and regulatory T cells that regulate homeostasis [75]. *Cryptosporidium* spp. infection is more severe (potentially fatal) and longer lasting in immunocompromised individuals with defective adaptive immune responses [76,77]. The crucial role of T-cell responses in *Cryptosporidium* spp. infection is obvious when studying HIV-infected patients [78] and patients with an immunodeficiency affecting T-cells [76].

**Figure 2:**
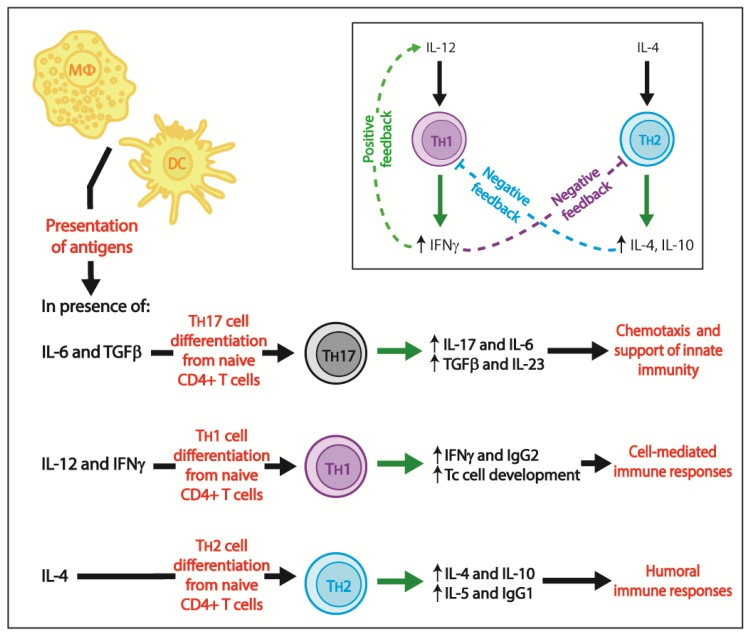
Adaptive immune responses during *Cryptosporidium* spp. infection. Green solid lines show release of molecules, green dotted line illustrates positive feedback, light blue and purple dotted lines represent negative feedbacks, black solid lines present direction of effects and red wordings define effects. MΦ = macrophage, DC = dendritic cell, T_H_1 = T_H_1 T cell, T_H_2 = T_H_2 T cell, T_H_17 = T_H_17 T cell.

As reviewed [60], CD4+ T cells are key actors in mounting adequate immune responses against cryptosporidiosis. Indeed, during the acute phase of infection involving innate immunity, CD4+ T cells are essential to clear *Cryptosporidium* spp. [19]. *Cryptosporidium* spp. infection is particularly frequent in AIDS patients with CD4+ T cell counts of <100 cells/μL [79]. CD4+ T cells counts <50 cells/µL are correlated with worse disease outcomes in immunocompromised patients [50,76,80,81,82]. T_H_17 cells constitute the first subset of CD4+ T helper cells to differentiate upon exposure of antigen-presenting cells to pathogens and are therefore important during the early stages of an infection [62]. T_H_17 cells differentiate from naive CD4+ T cells in presence of IL-6 and TGFβ (produced by dendritic cells), but in the absence of IL-12 and IL-4 [62] (Figure 2). IL-23 stimulates T_H_17 cells to produce IL-17, but not IFNγ or IL-4 [62]. Because IL-17 is involved in cytokine and chemokine secretion, which will have a chemotactic effect on neutrophils at the site of infection, IL-17 supports innate immunity against pathogens [62] (Figure 2). Among other T_H_17 cytokines, IL-17, IL-6, TNFα, TGFβ and IL-23 are found in increased levels in the gut-associated lymphoid tissue and spleen of immunosuppressed BALB/c mice infected with *C. parvum* [83] (Figure 2).

**Figure 3:**
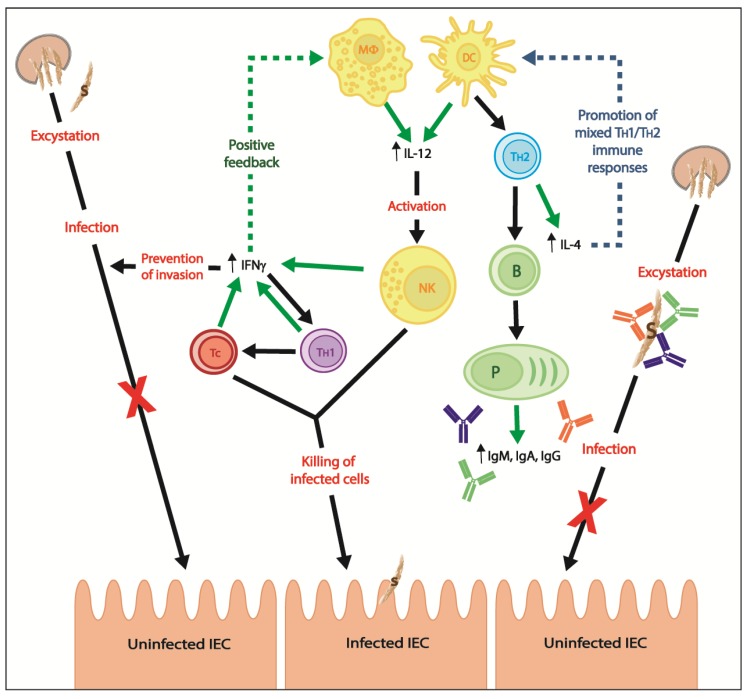
Protective immune responses during *Cryptosporidium* spp. infection and targets for vaccination. Green solid lines show release of molecules, green dotted line illustrates positive feedback, dark blue dotted line represents induction of mixed immune responses, black solid lines present direction of effects and red wordings define effects. MΦ = macrophage, DC = dendritic cell, NK = NK cell, T_H_1 = T_H_1 T cell, T_H_2 = T_H_2 T cell, Tc = cytotoxic T cell, B = B cell, P = plasmocyte, S = sporozoite and IEC = intestinal epithelial cell.

Promotion of cell-mediated immune responses and killing of infected cells resulted, in part, from macrophages and dendritic cells secretion of IL-12 and activated NK cells secretion of IFNγ [84] (Figure 3). In fact, IL-12 and IFNγ induce differentiation of naive CD4+ T cells to T_H_1 cells which will, among other effects, secrete IFNγ, produce IgG2 and promote differentiation of cytotoxic T cells from CD8+ precursors [84] (Figure 2). IFNγ has a positive feedback on IL-12 secretion by activating macrophages, while having a negative feedback on the T_H_2 differentiation of naive CD4+ T cells (Figure 2, insert); consequently, IFNγ strongly promotes a T_H_1 environment [84]. In contrast, IL-4 induces differentiation of CD4+ T_H_2 cells which, among other effects, induce production of IgG1, activate eosinophils and secrete IL-5, IL-4 and IL-10 (Figure 2); IL-4 and IL-10 have a negative feedback on T_H_1 cells [84] (Figure 2, insert). There is therefore a balance between T_H_1 and T_H_2 immune responses; cytokines secreted in a T_H_1 environment inhibit T_H_2 differentiation and vice versa. During *Cryptosporidium* spp. infection, CD4+ intraepithelial lymphocytes (IELs) produce IFNγ which is essential for innate immunity and adaptive T_H_1 immune responses and has a direct inhibitory effect on *Cryptosporidium* spp. development in host enterocytes [39,60].

The role of cytokines in *Cryptosporidium* spp. infection has been reviewed elsewhere [69]. Because of their importance in the immune response to *C. parvum* infection, they will briefly be reviewed here as well. As mentioned, IFNγ has a vital role in controlling early phase infection as a major component of the innate immune response. However, this proinflammatory cytokine also has an important role in adaptive immunity [69]. IL-12 and IFNγ promote development of naive CD4+ T cells into T_H_1 cells [69,84] (Figure 2) which contribute to the killing of intracellular microorganisms, such as *Cryptosporidium* spp., by stimulating phagocytosis, neutrophil degranulation, and release of reactive oxygen species [69,85,86,87]. In addition, IL-4 has a protective role in *Cryptosporidium* spp. infection via IL-4-induced differentiation of naive CD4+ T cells into T_H_2 cells [69] (Figure 2). In C57BL/6 adult mice, IFNγ-producing CD4+ T cells were essential in the initial phases of *C. parvum* infection to control the severity of infection, while IL-4-producing CD4+ T cells were important to accelerate resolution of infection [88]. Therefore, even if cytokines associated with T_H_1 immune responses (e.g., IFNγ and IL-12) are essential to clear *C. parvum* infection, some cytokines associated with T_H_2 immune responses (e.g., IL-4) have an important supporting role [89]. Wild-type, but not IFNγKO, mice treated with IL-4 neutralizing antibodies were less susceptible to *C. parvum* infection than untreated mice; IL-4 can therefore have an IFNγ-dependent protective role [89]. Thus, typical T_H_2 cytokines (i.e., IL-4) can potentially protect against cryptosporidiosis via T_H_1 immune responses [69] (Figure 3), as already reported for *Leishmania major* infection [90]. 

CD8+ T-cells are also important for clearance of the parasite. CD8+ T-cells also produce IFNγ in response to infection (Figure 3) and potentially lyse *Cryptosporidium* spp.-infected IECs through the secretion of anti-parasitic cytotoxic granules [91]. However, CD8+ T-cells are not major actors in adaptive immune responses against *Cryptosporidium* spp. infection. *C. parvum*-infected SCID mouse recipients of splenocytes from immunocompetent mice cleared infection unless treated with anti-CD4+ or anti-INFγ monoclonal antibodies, while anti-CD8+ monoclonal antibodies had no effect on the outcome [92]. SCID mice injected with IELs from immune BALB/c donors shed fewer oocysts and recovered more rapidly from *C. muris* infection; protection was abrogated by depletion of CD4+ T cells, but not CD8+ T cells, from IELs [93]. In addition, BALB/c mice infected with *C. muris* and treated with anti-CD4 monoclonal antibodies had increased duration of patent infection and oocyst shedding, while mice treated with anti-CD8 monoclonal antibodies had only a moderate increase in oocyst shedding [94].

### 3.2. Humoral Immune Responses

Although the important role of cell-mediated immune responses is well-described in *Cryptosporidium* spp. infection, the importance of humoral immune responses is not fully understood [35]. As part of mucosal immune responses, B-cells represent a major subset of GALT immunity [95] and gut resident B-cells undergo V(D)J recombination to produce secretory IgA [96,97]. Also, systemic *Cryptosporidium* spp.-specific antibodies, notably serum IgM, IgA and IgG, are generated following infection [98,99,100]. Generally, these antibodies are insufficient to prevent and control *Cryptosporidium* spp. infection [98] and are not essential for recovery and clearance of the parasite [101]. However, antibodies may play a supportive role in protection, as hyperimmune bovine colostrum (HBC) has undeniable prophylactic and therapeutic effects [102,103,104]. In fact, many studies report that administration of hyperimmune colostrum/antibodies protects newborn animals against *Cryptosporidium* spp. infection [19]. The ability of antibodies to prevent cryptosporidiosis has not been thoroughly characterized in human medicine and lessons learned from veterinary medicine will be reviewed here.

#### 3.2.1. Bovine Cryptosporidiosis and Colostrum-Treatment of Calves

As is true for human cryptosporidiosis, mucosal immune responses are also important for control of bovine cryptosporidiosis [105]. Lamina propria lymphocytes from *C. parvum*-infected calves express high levels of IFNγ and IgG1+, and IgG2+ B lymphocytes are present in ileal villi in infected calves [105]. Also, IL-10 expression was reported by IELs of *C. parvum*-infected calves [106]. Peripheral blood mononuclear cells from calves recovering from *C. parvum* infection show CD4+ T cell proliferation and IFNγ expression [107]. Antibody titers in experimentally-infected calf serum peak 9 days post-infection (coinciding with the peak of oocyst shedding) and remain high thereafter [108]. Fecal IgM and IgA titers of experimentally-infected calves also peak 10 days post-infection (2 days after the peak of oocyst shedding) [109]. In another study, fecal IgM, IgA and IgG titers peaked 14 days post-infection and IgA titers remained high for at least 30 days post-infection while IgM and IgG titers decreased quickly [110]. Fecal antibody titers tend to raise when oocyst shedding increases and oocyst shedding stops when antibody titers peak [110].

Among *C. parvum* sporozoite surface proteins, p23 is one of the most immunogenic. Anti-p23 antibodies (IgM, IgA, IgG1 and IgG2) were detected in feces of *C. parvum*-experimentally-infected calves [111]. In that study, one calf which did not excrete detectable fecal anti-23 antibodies died of infection and another calf with pre-existing anti-p23 IgM antibodies did not shed oocysts [111]. Another study presented similar results for a calf with pre-existing anti-p23 antibodies [112]. In clinically normal newborn calves, anti-p23 IgM, IgA, IgG1 and IgG2 antibodies were detected in feces via passive transfer from colostrum [113], suggesting a maternal source of pre-existing anti-p23 antibodies.

Because newborn calves can get infected as soon as their day of birth [114], a promising approach against bovine cryptosporidiosis is vaccination of pregnant cows to engender production of HBC that will protect newborn dairy calves against *C. parvum* [115]. p23 is a promising antigen for vaccination against bovine cryptosporidiosis. HBC was produced by pregnant cows vaccinated using p23 and *C. parvum*-challenged HBC-treated calves had no diarrhea and oocyst shedding was reduced by 99.8% [115]. In another study, HBC-treated calves showed delayed oocyst shedding with more than 90% reduction in oocysts shed [116]; also, no clinical sign of cryptosporidiosis was reported in the HBC-treated calves [116].

#### 3.2.2. Antibody Treatment of Immunocompromised Mice

Several studies showing the ability of monoclonal antibodies to partially reduce oocyst shedding or intestinal parasite burden in immunocompromised mice support the importance of antibodies for protection against cryptosporidiosis. Oral gavage of *C. parvum*-infected SCID mice with an anti-SA-1 (*C. parvum* surface antigen-1) [117] IgM monoclonal antibody (mAb) reduced oocyst shedding [118]. A neutralizing anti-CSL (another *C. parvum* sporozoite ligand) mAb delivered by oral gavage reduced infection (as well as combination of mAbs raised against P23, GP25-200 and CSL) of adult IFNγ-depleted SCID mice [119]. IgA mAbs specific for P23 *C. parvum* surface protein passively immunized neonatal BALB/c mice and reduced intestinal parasite burden by up to 72% [120]. Oral treatment of SCID mice with anti-*C. parvum* IgY egg yolk antibody reduced parasite shedding [121]. An antibody-rich fraction extracted from HBC from cows immunized with *C. parvum* sonicated oocysts or sporozoites given orally to adult SCID mice resulted in reduced oocyst shedding and intestinal parasite burden [122]. Using hyperimmune ovine colostral whey, the intensity of infection in newborn NMRI mice was inversely proportional to the amount of antibody administered and number of doses [123]. Therefore, mAbs or hyperimmune colostrum might be an option for therapy of human cryptosporidiosis.

#### 3.2.3. Treatment of Immunocompromised *Cryptosporidium* spp.-Infected Patients with Hyperimmune Bovine Colostrum

It is important to note that the importance of *Cryptosporidium* spp.-specific antibodies for protection against cryptosporidiosis might not be equal between humans and animal models. For example, high levels of fecal *C. parvum*-specific IgA and IgM antibodies following infection correlate with reduced oocyst shedding in *C. parvum-*infected athymic C57BL/6 nude mice [124]. IgA antibodies are present in *Cryptosporidium* spp.-infected AIDS patients, but this response is insufficient to protect against cryptosporidiosis [98,125]. In other words, anti-*C. parvum* antibodies alone cannot clear infection in immunocompromised *Cryptosporidium* spp.-infected patients without the support of CD4+ T cells [125]. Therefore, conclusions drawn from immunocompromised mouse models may not always be applicable for immunocompromised humans. In fact, contradictory results are reported in the literature. On one side, some studies suggest a partial protective role of antibodies from HBC against cryptosporidiosis in immunocompromised patients [102,126,127] and HBC in concentrate powder form was an effective therapeutic approach in *C. parvum*-infected HIV patients as it significantly decreased stool weight and frequency [128]. On the other side, two studies showed that only some patients had reduced oocyst shedding after treatment [129] and that HBC had no protective effect compared to placebo to decrease stool volume or oocyst shedding [130].

## 4. Vaccines against *Cryptosporidium* spp. Infection

### 4.1. DNA Vaccines and Subunit Vaccines

Many types of vaccines exist, such as DNA vaccines, subunit vaccines, live-attenuated vaccines and vector vaccines [131,132]. Many promising vaccine approaches for cryptosporidiosis have been reviewed elsewhere [19,35,101]; briefly, some DNA and subunit vaccine candidates will be reviewed here. DNA vaccines encoding some surface proteins of *C. parvum* (such as Cp12 and Cp21 [133] or cp15 and p23 [134] or CP15/60 [135]) lead to protective immune responses via production of high IgG levels [133,134], elevated T_H_1 cytokines [134] and/or increase in the numbers of CD4+ and CD8+ T cells [133]. Protection from DNA vaccines resulted in up to 77.5% reduction in oocyst shedding after challenge [133,134].

Subunit vaccines have been commonly used in vaccine development against cryptosporidiosis and several immunodominant proteins have been identified as potential vaccine candidates [136]. As mentioned previously, pregnant cows were vaccinated with *C. parvum* sporozoite p23 surface protein and resulting HBC was protective for *C. parvum*-challenged calves [115]. Also, in another study, anti-P23 HBC-treated calves showed no clinical sign of cryptosporidiosis and reduced and delayed oocyst shedding [116]. HBC from pregnant cows immunized with CP15/60 recombinant protein successfully transferred antibodies to calves via colostrum intake; however, challenge of treated calves was not presented [137]. In mice, divalent recombinant Cp15-23 led to significant antibody and T_H_1 cytokine production and elevated numbers of CD4+, but did confer only partial protection against *C. parvum* challenge [138].

### 4.2. Live-Attenuated Vaccine

Live-attenuated vaccines have historically been shown to be best at eliciting long lasting memory immune responses, whereas subunit vaccines elicit a more modest memory response, often requiring subsequent booster doses to achieve long lasting immunity [132]. Attenuated vaccines were first developed for viral and bacterial pathogens because of the inherent complexity of parasitic organisms; however, some vaccine development is ongoing for a few pathogenic parasites [139].

Live-attenuated vaccines elicit strong T_H_1 biased immune responses and offer protective cell-mediated immunity [132]. Several live-attenuated vaccines have been developed against protozoan parasites causing enteric disease, i.e., *Eimeria* [140,141]. Early studies showed that chickens receiving irradiated *E. maxima* oocysts were protected against coccidiosis-induced weight loss [142]. Also, live-attenuated *Toxoplasma gondii* induced protective immunity against toxoplasmosis in sheep for at least 6 months [143,144]. In addition, live vaccines against another parasite, *Leishmania* spp., have recently been studied [145]. A non-pathogenic species, *L. tarentolae*, elicits strong protective T_H_1 immune responses in mice against *L. donovani* [146]. Similar responses were observed in mice vaccinated with attenuated *L. donovani* [147]. Although this approach is promising, a live-attenuated vaccine may not be ideal for cryptosporidiosis due to its host requirement for replication [19]. This is further exacerbated by the lack of a continuous in vitro culture system allowing oocyst production for *Cryptosporidium* spp. [148]. Nonetheless, γ-irradiation has been used on *Cryptosporidium* spp. oocysts or sporozoites to reduce their viability and infectivity [149]. Irradiated *C. parvum* oocysts were shown to elicit protective immune responses in calves challenged at 3 weeks post-vaccination [150].

### 4.3. Vaccine Vectors

Vaccine vectors came into play in the early 1990s [151], but the first vaccine vector to be licensed is a chimeric yellow fever attenuated strain in 2010 [152]. A vaccine against *Cryptosporidium* spp. should stimulate mucosal immune responses by promoting uptake of antigens by microfold cells (M cells), specialized epithelial cells adjacent to enterocytes that facilitate the passage of antigens to Peyer’s patches [62]. Intestinal antigen delivery to the M cells could be achieved using a vaccine delivery system such as attenuated bacterial or viral vectors [153,154]. To our knowledge, no viral vectors have been used in candidate vaccines for *Cryptosporidium* spp., but several live bacterial vectors have been studied [155,156,157].

Bacterial vaccine vectors are very promising for vaccine antigen delivery as they can elicit protective immune responses against bacterial, viral and protozoan pathogens in both mice and humans [158]. For instance, delivery of influenza hemagglutinin and neuraminidase using an attenuated *S. typhimurium* vector induced strong protective cellular and humoral immunity against Influenza A virus [159]. Also, delivery of *Trichinella spiralis* DNA using an attenuated *S. typhimurium* elicited protective mixed T_H_1/T_H_2 immune responses in mice [160]. Moreover, *Plasmodium falciparum* tCSP genes fused to secretion signals were delivered through *S. typhimurium* and boosted with a DNA vaccine and elicited strong cellular T_H_1 immune responses [161]. Overall, the many advantages of this vaccine approach (ease of administration and low production cost) engender excellent candidates for vaccine development [162]. Fusing the protein of interest to a secretion signal and a chaperone binding domain of *S. enterica* allows secretion of the antigen of interest through the type III secretion system-dependent for delivery to antigen-presenting cells [163]. 

A number of attenuated *S. typhimurium* vectors expressing *Cryptosporidium* spp. antigens have been generated [155,156,157]. Promising humoral and cellular immune responses were obtained from a prime boost technique with *Salmonella enterica* serovar Typhi CVD-908-*htrA* and cytolysin A (ClyA) fused to either *C. hominis* apyrase (CApy), profilin or Cp15 [155]. In mice, these vaccines elicited strong humoral immune responses with high production of IgG1 and IgG2b and interesting cellular immune responses via production of different levels of several cytokines (IFNγ, IL-2, IL-6, and IL-12) [155]. Attenuated *Salmonella enterica* serovar Typhimurium vaccine strain SL3261 expressing *C. parvum* Cp23 or Cp40 fused to fragment C of tetanus toxin elicited humoral immune responses when delivered as an oral boost after subcutaneous immunization with cp23 or cp40 DNA [156]. An attenuated *Salmonella enterica* serovar Typhi CVD 908-*htrA* expressing Cp15 delivered intranasally in mice showed high production of IL-6, IFNγ and Cp15-specific IgG [157]. However, vaccination did not result in protection against *C. parvum* infection in mice [157]. Another vector system used for *Cryptosporidium* spp. antigen delivery is *T. gondii* [164]. Immunization of mice with *T. gondii* expressing *C. parvum* P23 antigen resulted in high levels of serum IgG, predominantly IgG1, which is characteristic of a T_H_2 immune response [164,165]. In another study, *Lactobacillus casei* Zhang (a probiotic bacterium [166]) was used to deliver *C. parvum* P23 to mice and generated increased levels of IFNγ, IL-6, serum IgG and fecal IgA [167].

Overall, vaccine vectors show promising immunological results and appear to be an interesting option for vaccine development against cryptosporidiosis. Although one challenge study showed no protection after vaccination [157], more studies using various vectors and immunogens are needed to assess the true potential of this method. It will be very interesting to determine if they show better protection against *Cryptosporidium* spp. infection than their non-vector strategies. The high carrying capacity of vectors is also an advantage, as they can deliver multiple antigens and even adjuvants to the target site [168]. Vaccine vectors can also be used either alone or in combination with DNA or antigen-based vaccine candidates as a ‘prime-pull’ method [155,161].

### 4.4. Prime-Pull Vaccine Approach

The prime-pull vaccine approach primes the immune system with an antigen to elicit strong systemic T cells immune responses and then ‘pulls’ T cell immune responses at the site of infection using local delivery of immunogens and/or pro-inflammatory molecules to elicit local protective and long-lasting memory responses [169]. In other words, the ‘prime’ immunization using intramuscular delivery of antigen(s) elicits systemic T cell immune responses while the ‘pull’ immunization allows for the formation of a strong pool of tissue-resident T cells [169]. As mentioned above, the prime-pull approach has been used in combination with vaccine vectors against *Cryptosporidium* spp. infection in various delivery schedules and methods [155,156,157]. In some studies, *C. parvum* DNA was used to ‘prime’ mice and the *C. parvum* antigen-expressing *Salmonella* spp. vector was given as a boost [156]. In other studies, the *Salmonella* spp. vector vaccine was given as a ‘prime’ and then boosted with recombinant protein given intraperitoneally [155,157]. Overall, the prime-pull method elicits much stronger immune responses than the vector or the antigen alone [155,156,157].

## 5. Conclusions and Future Directions

As *Cryptosporidium* spp.-infected immunocompetent individuals only present with transient diarrhea while immunocompromised patients and infants in developing countries can have very severe and life-threatening cryptosporidiosis, the competency of the host immune system to raise adequate immune responses is the key factor to clear *Cryptosporidium* spp. parasites. The pathogenesis of cryptosporidiosis is incompletely understood because this protozoan parasite induces complex host immune responses. Innate immunity can contain *C. parvum* infection via the action of IL-18- and IL-12-activated macrophages and NK cells which induce NK cells-dependent and NK cells-independent IFNγ production (Figure 1 and Figure 3). Adaptive immunity will clear *C. parvum* infection via the action of CD4+ T_H_1 cell-mediated immune responses which induce IFNγ production and killing of infected IEC; T_H_2 immune responses and humoral immunity have a non-negligible supportive role (Figure 2 and Figure 3).

To sum up, protective immune responses against *Cryptosporidium* spp. infection require strong mucosal T_H_1 cell-mediated immune responses with the support of a T_H_2-dependant *Cryptosporidium* spp.-specific humoral immunity (Figure 3). A vaccine that induces such immune responses, if safe for use in children and immunocompromised individuals, should be the best candidate to prevent cryptosporidiosis. Furthermore, because *Cryptosporidium* spp. infects the intestinal epithelia, a vaccine against cryptosporidiosis would ideally elicit strong mucosal immune responses [35]. Vaccine vectors using the ‘prime-pull’ approach represent a new era in vaccine development and we believe that these new techniques have the potential to elicit more targeted immune responses and localized protection against *Cryptosporidium* spp. infection [156]. Results from ongoing studies will determine potential of this new vaccine approach against *Cryptosporidium* spp. infection.
